# Bronchoscopic management of airway foreign bodies in adults: a narrative educational review

**DOI:** 10.3389/fmed.2026.1779715

**Published:** 2026-03-04

**Authors:** Jing Chi, Yang Bai

**Affiliations:** Department of Respiratory and Critical Care Medicine, The First Affiliated Hospital of Chongqing Medical University, Chongqing, China

**Keywords:** airway foreign body, bronchoscopic management, interventional pulmonology, narrative review, postgraduate education

## Abstract

**Objective:**

Airway foreign body (AFB) in adults remains a potentially life-threatening emergency, which lacks standardized clinical pathways of management. This review aims to synthesize current evidence on the clinical recognition, imaging work-up, and bronchoscopic management of AFB in adults and to propose a practical, stepwise algorithm, enabling interventional pulmonologists to establish a systematic retrieval framework as early as possible and thereby optimize care for adult patients.

**Methods:**

A narrative review was conducted by searching PubMed for studies focusing on the bronchoscopic management of AFB in adults. The emphasis is on the tool selection, critical techniques, and procedural nuances for AFB retrieval. Personal clinical experience also informed the interpretation and contextualization.

**Results:**

Adult AFB typically presents with chronic cough, dyspnea, wheeze, or recurrent post-obstructive pneumonia. Computed tomography (CT) is the first-line imaging modality (pooled sensitivity 98.8%, specificity 96.6%), but radiolucent organic material may yield false-negative results; therefore, high clinical suspicion warrants direct bronchoscopy. The right main bronchus is the most common site because of its anatomical features. Pre-intervention planning matches AFB characteristics (site, size & shape, and substance) with patient status to decide between rigid and flexible scopes and to select retrieval accessories. Flexible bronchoscopy under general anesthesia via laryngeal mask airway achieves > 90% success in adults, which is preferred for peripheral items, whereas rigid bronchoscopy remains the gold standard for large, sharp, or proximal AFBs. Tool choice follows an object-specific strategy: forceps for metal/bone, snare for bulky, irregular items, basket for smooth, round seeds, balloon for impacted distal AFBs after dilation, and cryoprobe for semisolid, water-rich material (blood clot, mucus plug, food). Complication rates are lowest when dislodgement and extraction are performed as a single, controlled maneuver under continuous visualization; hybrid rigid–flexible approaches further improve safety.

**Conclusions:**

Consider AFB in adults with unexplained chronic respiratory symptoms. CT guides but does not replace bronchoscopy. An individualized strategy—flexible scope first (in patients with stable status), rigid scope reserved for selected complex cases—combined with object-tailored tools optimizes successful AFB retrieval while minimizing morbidity. Maintaining both rigid and flexible systems, trained multidisciplinary teams, and strict manometric monitoring are essential components of AFB retrieval.

## Introduction

Airway foreign body (AFB), whether endogenous or aspirated, is a potentially life-threatening emergency, especially among vulnerable pediatric and elderly patients (1, 2). Common risk factors for aspirated AFBs in adults encompass impaired swallowing coordination, reduced cough reflex, altered mental status, intoxication with alcohol or drugs, neuromuscular weakness, poor dentition, and recent dental, pharyngeal, or airway procedures (3–7). These risk factors frequently hinder patients from reporting aspiration incidents (8). The clinical manifestation of AFB in adults, which typically results from distal airway obstruction, generally differs from that observed in children (9). AFB in adults primarily presents with nonspecific chronic or subacute respiratory symptoms, such as cough, dyspnea, hemoptysis, fever, and chest pain, which overlap extensively with other pulmonary diseases (10). Some patients are completely asymptomatic, with AFB only found incidentally during routine check-ups. Abnormal opacities and pulmonary infiltrates represent the predominant imaging findings in elderly patients with AFB (8, 11). This unrecalled aspiration, symptom overlap, and non-specific imaging findings often lead to the misdiagnosis of AFB as asthma, bronchitis, conventional pneumonia, or even lung cancer in adults (12–14). The prevalence of AFB among adults with unexplained chronic respiratory symptoms is rare, as reflected by the bronchoscopy data. Among 25,998 flexible bronchoscopies performed over 35 years, only 65 patients (0.25%) underwent the procedure for clinical suspicion of AFB (15). A systematic review confirmed this rarity: the pooled proportion of flexible bronchoscopies performed for AFB among total flexible bronchoscopies was 0.24% (15).

Bronchoscopy, providing direct visualization and intervention, is the necessary procedure in diagnosing and retrieving AFBs (15, 16). The decision between flexible and rigid bronchoscopy should be made based on several considerations, including the specific AFB features, the patient's status, the physician's expertise, and the equipment accessibility (17). Flexible bronchoscopy is usually favored in adults due to its wide accessibility, convenience of use, and ability to be conducted without general anesthesia (18). Nevertheless, there are cases when rigid bronchoscopy is still preferred since it allows for superior visualization and utilization of larger tools for more effective AFB retrieval (19).

Most available data are derived from small, single-center case series, which lack a standardized, procedure-centric roadmap aligned with the realities of contemporary interventional practice for AFB management in adults. This narrative review synthesizes current clinical evidence and addresses critical gaps in diagnostic strategies, imaging work-up, bronchoscopic modality selection, and interventional tool utilization, in combination with clinical cases, which establishes a practical, evidence-based framework to optimize AFB retrieval in adults.

## Methods

A narrative review was conducted by searching PubMed (up to October 2025) for relevant studies on AFB management (Table 1), regarding clinical recognition, imaging work-up, bronchoscopic selection, and interventional tool utilization in adults, adhering to the instructions for writing narrative literature reviews for peer-reviewed journals (20). The inclusion criteria prioritized evidence that was clinically actionable and specific to AFB in adults. The restriction to core topics (clinical presentation, CT performance, bronchoscope choice, and tool selection) was consistent with the review's objective of establishing a systematic, procedure-centric framework. The restriction to English-language publications was pragmatic, ensuring consistent access and precise interpretation through PubMed's comprehensive global coverage. Methodological rigor was improved by adhering to peer-reviewed narrative review guidelines. Study inclusion required that they (1) focused on patients with confirmed or suspected AFB, especially in adults; (2) addressed at least one core review topic (clinical presentation, CT diagnostic performance, bronchoscope selection, or interventional tool features and technique); and (3) were published in English.

**Table 1:** The search strategy.

**Items**	**Specification**
Database searched	Pubmed
Search terms used	“Airway Foreign Body” [MeSH] OR “Foreign Body Aspiration” [MeSH] OR “AFB”) AND (“Adult” [MeSH] OR “Adults”) AND (“Bronchoscopy” [MeSH] OR “Rigid Bronchoscopy” OR “Flexible Bronchoscopy” OR “Retrieval Tool”
Timeframe	Upto October 2025
Inclusion criteria	Articles were included if they were written in English and met the following criteria: (1) focused on patients with confirmed or suspected AFB; (2) addressed at least one core review topic (clinical presentation, CT diagnostic performance, bronchoscope selection, or interventional tool features and technique).
Selection process	The selection process was conducted by Yang Bai and Jing Chi

## Results

### Clinical presentation

Unlike the more severe symptoms reported in children (21), the clinical manifestation of AFB in adults is frequently insidious and nonspecific (22). Chronic cough is the predominant symptom in adults, occurring in 60% to 85% of cases (5, 22, 23). Dyspnea, unilateral wheeze, hemoptysis, and repeated same-lobe pneumonia are other symptoms that may occur. These symptoms may not be recognized promptly as indicative of AFBs (24). The reason for this late detection is, in part, that the AFB often affects the distal airways, resulting in mild symptoms that might be misinterpreted as other respiratory disorders, such as asthma, bronchitis, conventional pneumonia, or even lung cancer (12–14). Obstructive pneumonia, chronic inflammation, and bronchiectasis are among the potential complications that arise from a delayed diagnosis when adult patients cannot recall the choking episode or aspiration event (25, 26). This situation illustrates the importance of considering AFB in the differential diagnosis of persistent respiratory symptoms, especially in those with risk factors for AFBs, even in the absence of a clear history of choking and aspiration (4–6).

### Imaging and anatomical considerations

**Figure 1:**
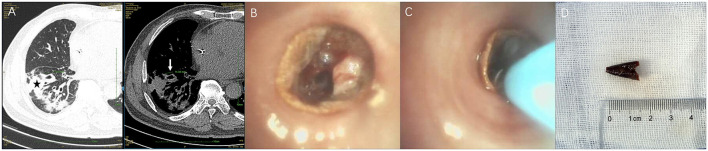
Radiolucent organic AFB, RLL. **(A)** CT: saccular bronchial dilation (white arrow, left) and adjacent consolidation (black asterisk, right). **(B)** Thin bronchoscopy: chili-pepper tip lodged in anterior RLL. **(C)** Cryoprobe inserted for extraction. **(D)** Retrieved 1.5 cm chili-pepper tip matching the dilated segment on CT. AFB, airway foreign body; CT, computed tomography; RLL, right lower lobe.

Computed tomography (CT) has become the standard for identifying AFBs in the airway, demonstrating exceptional diagnostic accuracy with a pooled sensitivity of 98.8% and specificity of 96.6% (27). While high-resolution CT (widely used in obstructive respiratory disorders and bronchiectasis) offers thin-slice scanning for small structures and subtle airway changes, standard multidetector CT is sufficient to visualize AFBs (including these low-attenuation intrabronchial materials), to detect complications (such as atelectasis, hyperinflation, and pneumonia), and to provide 3D reconstructions for procedural planning (28). In adults, the AFB itself is visualized directly on CT imaging far more often than in pediatric cases, in whom radiolucent objects and narrower airways typically generate only indirect evidence—regional hyperinflation, atelectasis, post-obstructive pneumonia, bronchiectasis, or pleural effusion (11). It is important to note that certain organic AFBs (e.g., meat, plant matter, bread, or shrimp) may remain radiolucent even when evaluated via multidetector CT (29). Therefore, if there is a high clinical suspicion of an AFB but the CT fails to show positive findings, a bronchoscopy is necessary to confirm or rule out its presence, even with the unrecalled aspiration. Figure 1 presents a case of a radiolucent chili-pepper tip lodged in the anterior right lower lobe. Additionally, CT aids in delineating the precise location of AFBs with greater detail (site, size, shape, and substance) and enhances the sensitivity for detecting radiolucent AFBs and AFB-related complications (30). The tracheobronchial anatomy predisposes aspirated AFBs to more often get lodged in the right main bronchus. This phenomenon is ascribed to the right main bronchus being shorter and larger, forming a more vertical angle with the trachea in comparison to the left (5, 8, 31). Recognition of this anatomical tendency is essential to design effective diagnostic imaging strategies and bronchoscopic interventions.

### Pre-intervention planning

The presumed site, size & shape, and substance of the AFBs, in combination with the status of the patients, should facilitate the formulation of a comprehensive plan before the bronchoscopic intervention. This pre-intervention strategy includes selecting an appropriate anesthesia method, a suitable rigid or flexible bronchoscope, and the optimal interventional tools for AFB retrieval. When deciding on a retrieval tool and a procedure, it is important to consider the AFB's size, shape, and substance. For instance, a large AFB lodged in the main bronchus may necessitate a rigid bronchoscope, of which the wide lumen permits secure grasping and extraction while avoiding damage to the glottic structures (32). A spherical AFB with a smooth surface—such as beans, peanuts, or almonds—may need a wire basket, since its slippery contour lacks edges or ridges for standard forceps to grasp. The basket can encircle the item, distribute pressure uniformly, and cradle it firmly—preventing slippage or shearing during passage through the airway (33). A semisolid AFB—such as blood clots or inspissated mucous plugs—may necessitate cryoextraction, because its high fluid content renders it too friable for forceps or baskets to seize (34). By freezing the probe tip to −20 °C or lower, the interstitial water crystallizes and fuses the entire mass to the metal surface, transforming the fragile slurry into a single rigid “popsicle” (35). This ice-clot cylinder can then be withdrawn en bloc, minimizing distal embolization, sparing the mucosa, and restoring ventilation in one controlled maneuver (35). Moreover, the patient's clinical status and the potential need for general anesthesia must be assessed. The comprehensive pre-intervention planning is vital to guarantee successful AFB retrieval and minimize procedural risks.

### Rigid vs. flexible bronchoscopy

The selection between rigid and flexible bronchoscopy is determined by various factors. Rigid bronchoscopy remains the gold standard for AFB retrieval for its superior visualization, capacity to control bleeding, ability to utilize larger forceps and other tools, and the optical ventilation control, particularly in small children (36). However, rigid bronchoscopy necessitates general anesthesia and specialized expertise, and it is associated with an increased risk of complications, such as dental, glottic, or airway injury, as well as hypoxemia and hypercapnia (37). In many centers, the use of the rigid bronchoscope is further hampered by limited clinical experience, unavailable equipment, insufficient anesthetic support, and the absence of standardized protocols (38, 39). The unfavorable airway anatomy also obstructs the insertion of a rigid bronchoscope in some individuals (40). Flexible bronchoscopy, delivered through a laryngeal mask under general anesthesia, has emerged as a reliable alternative in adults, with a pooled success rate of 89.6% (95% confidence interval 86.1% to 93.2%) for AFB retrieval (15). While initially established in pediatric meta-analyses (41), the equivalent efficacy and comparable safety profiles of flexible versus rigid bronchoscopy for AFB retrieval extend similarly to adults, given the shared mechanisms underlying both modalities. The primary advantage of the flexible bronchoscope lies in its capacity to conduct a thorough evaluation of the peripheral airways, which are typical sites of aspirated AFBs in adults (3, 5). Moreover, the tool's adaptability allows for the removal of distal granulomas often associated with chronic AFBs and facilitates the detection of remnant pieces, thereby making it effective for managing peripherally lodged items (42). Flexible bronchoscopy also provides benefits in decreasing the desaturation risk and hospitalization length, while rigid bronchoscopy reduces the incidence of initial procedural failure and conversion rates compared to flexible bronchoscopy (41). Consequently, flexible bronchoscopy might be regarded as the first-line option for AFB retrieval in adults, with rigid bronchoscopy held in reserve for initial failure and selected complex cases with respiratory failure or hemodynamic instability, as well as for large or sharp AFBs, where robust airway control and retrieval efficacy are critical (23, 43–45). The combined approach can be used when circumstances dictate (43, 46).

These results endorse an individualized strategy for bronchoscopy selection, guided by patient anatomy, AFB features (site, size, shape, and substance), and operator expertise. Rigid bronchoscopy may be favored for proximal, sharp, or large AFBs (to prevent conversion from flexible bronchoscopic failure), while flexible bronchoscopy is appropriate for distal AFBs or patients with an elevated risk of desaturation. The complementary nature of the two techniques underscores the need for them to be available in clinical settings. The combination of rigid and flexible bronchoscopy ensures patient safety and procedural success.

### Tools and techniques for AFB retrieval

The essentials of AFB retrieval in the airway hinge on two steps: (1) atraumatic dislodgement of the object from its initial lodging site and (2) careful trans-airway withdrawal. Therefore, all tools for AFB retrieval can be categorized into two groups: those that fulfill only the first step and those that accomplish both steps simultaneously (Table 2). Representative cases illustrating the application of these tools are shown in the following figures.

**Table 2:** Trans-airway foreign body retrieval: tool selection and phase-based extraction techniques.

**Interventional tools**	**Phase 1: atraumatic dislodgement**	**Phase 2: trans-airway withdrawal**	**Typical clinical notes**
Standard biopsy forceps	Direct grip on AFB surface or edge, followed by twisting to dislodge	Same forceps maintain grip during retrieval	Best for small, sharp AFBs; removing obstructive granulation tissue; risk of mucosal tear if excess torque
Rat-tooth flexible forceps			Best for median, sharp AFBs; risk of mucosal tear if excess torque
Rubber tip grasping forceps			Best for sharp, slippery AFBs; risk of mucosal tear if excess torque
Alligator flexible forceps			Best for large, flat AFBs; risk of mucosal tear if excess torque
Snare	Direct loop and tightening of AFB, followed by extraction to dislodge	Same snare keeps AFB ensnared during retrieval	Monopolar mode can coagulate surrounding granulation first; best for large AFBs beyond the forceps size
Basket	Basket opened distally, abutted, and cage AFB, followed by extraction	Same basket keeps AFB encircled during retrieval	Best for round AFBs; AFB collapses to radial compression for tight subglottic passage
Balloon	Balloon inflation distal to AFB followed by extraction, enables retrieval	Not designed for the procedure; requires adjunct tools except for AFB with lumen	Different sizes available; saline, not air, for inflation to avoid barotrauma
Cryoprobe	Freeze-adherence to semisolid water-rich AFB	Same probe with frozen tip during retrieval	Best for semisolid water-rich AFBs; needs CO_2_ or N_2_O; avoid if active bleeding

### Forceps

Flexible forceps remain the cornerstone for bronchoscopic retrieval of AFBs. They are highly effective for metallic or hard organic objects (such as pins, bone fragments, and dentures), which are the most commonly encountered in adult patients (15). Used in conjunction with flexible bronchoscopy (the preferred initial approach in most centers), flexible forceps enable successful extraction in 89.6–91.8% of cases, with minimal complications (15). Once a proximal AFB has been located, the appropriate forceps are applied to grasp it through the flexible bronchoscope, which is then withdrawn as a single unit. Forceps have various jaw configurations, allowing the endoscopist to tailor device selection to the object being retrieved (47). Standard biopsy forceps are generally used to extract small, slender metallic objects such as pins or needles, providing precise jaw alignment and adequate grip for these diminutive AFBs. Distal bronchial AFBs are often masked by excessive granulation tissue under bronchoscopy; in most published failures, the object could not even be visualized (32). Standard biopsy forceps can overcome this limitation by debriding the granulation tissue until the hidden AFB edge becomes visible, then grasping and withdrawing. Forceps with a distal tooth (rat-tooth or shark-tooth) with enhanced jaw capacity and grip strength offer a more secure grasp on the object being retrieved. Forceps with longer jaws (alligator-jaw) may occasionally be useful, especially for large, flat, hard objects (48, 49). Rubber-tip forceps with anti-slip grips are designed for small, sharp objects such as needles, pins, and blades (50). The recommended technique for AFB retrieval using forceps involves directly grasping the visible edge and subsequently executing gentle twisting or rocking motions to dislodge the item. The same forceps should maintain a secure grasp throughout the extraction. When extracting a sharp AFB, the tool should be applied to its pointed extremity so that the sharp margin trails during withdrawal, thereby minimizing the risk of laceration to the airway mucosa or vocal cords (51, 52).

**Figure 2:**
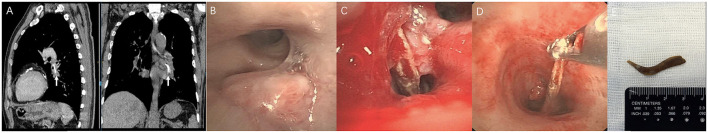
Impacted linear bone, LLL. **(A)** CT: linear hyperdensity (white arrow). **(B)** Bronchoscopy: granulation at LLL orifice. **(C)** Bone fragment exposed after standard biopsy forceps debridement. **(D)** Forceps grasped sharp edge for extraction. **(E)** 3 cm bone fragment. LLL, left lower lobe; CT, computed tomography.

Figure 2 presents a case of an impacted linear bone fragment in the left lower lobe (LLL). CT identified a linear hyperdensity (Figure 2A). Bronchoscopy showed granulation tissue at the LLL orifice (Figure 2B). After debridement with standard biopsy forceps, the bone fragment was exposed (Figure 2C), grasped by its sharp edge (Figure 2D), and extracted as a 3 cm-length fragment (Figure 2E).

### Snare

**Figure 3:**
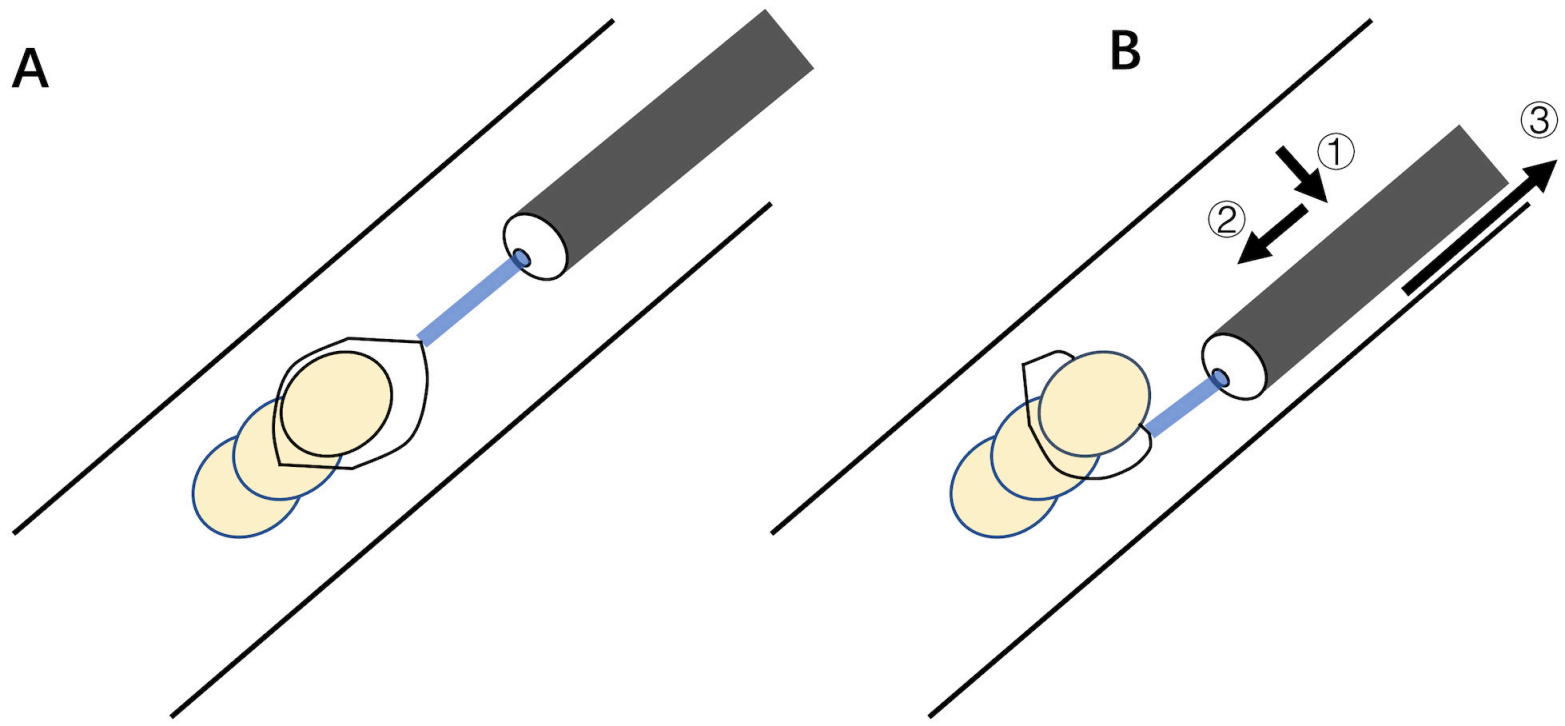
Schematic snare-loop technique for large rigid AFB. **(A)** Open snare into a sufficiently large loop parallel to AFB's long axis. **(B)** Advance scope to abut AFB (①), retract it downward to position the snare loop nearly perpendicular to AFB (②), and tighten loop for extraction (③). AFB, airway foreign body.

A wire-guided snare is a medical device typically composed of a flexible cable with an adjustable loop at its distal tip. The snare can be advanced through the working channel of a flexible bronchoscope, offering enhanced maneuverability in complex anatomical scenarios. Many types of snares, differing mainly in the size of the open loop, are available for endoscopic AFB retrieval (47). The technical tip for encircling the snare loop around the AFB is to open the snare to form a sufficiently large loop parallel to its long axis. The bronchoscope is carefully maneuvered to allow the loop to abut and downward to form a nearly perpendicular angle between the loop and the AFB (Figure 3). Then the AFB is secured by the tightened loop for extraction. The single motion allows secure grasping of large, rigid, or irregularly shaped objects (such as dental prostheses or broncholiths) more effectively than biopsy forceps or wire baskets, facilitating smooth extraction through the glottis as a single unit with the flexible bronchoscope (53, 54). It is less suitable for small or round objects that are difficult to loop. Furthermore, the presence of significant granulation tissue or airway inflammation can make snare application more challenging. In such cases, standard, cup- or spike-jaw biopsy forceps can be employed to clear the overgrown tissue and expose the edge of the AFB, thereby enabling subsequent grasping and retrieval (32). A multidisciplinary approach incorporating rigid and flexible bronchoscopy and the wire snare permits the successful extraction of difficult AFBs in the airway (55).

**Figure 4:**
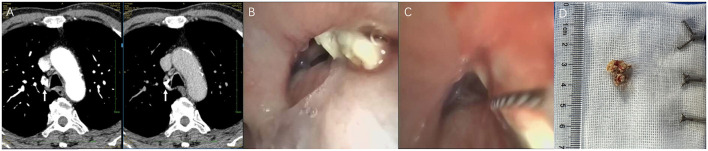
Impacted broncholith at RUL orifice. **(A)** CT: hyperdense focus (white arrow). **(B)** Bronchoscopy: broncholith wedges at RUL orifice. **(C)** Snare deployed after different forceps failed. **(D)** Retrieved 1 cm broncholith. RUL, right upper lobe; CT, computed tomography.

Figure 4 demonstrates the use of a snare for retrieving an impacted broncholith at the right upper lobe (RUL) orifice. CT showed a hyperdense focus at the RUL origin (Figure 4A). Bronchoscopy revealed a broncholith wedged at the RUL orifice (Figure 4B). After different forceps failed to grasp the object, the snare was successfully deployed (Figure 4C), retrieving a 1 cm broncholith (Figure 4D).

### Basket

**Figure 5:**
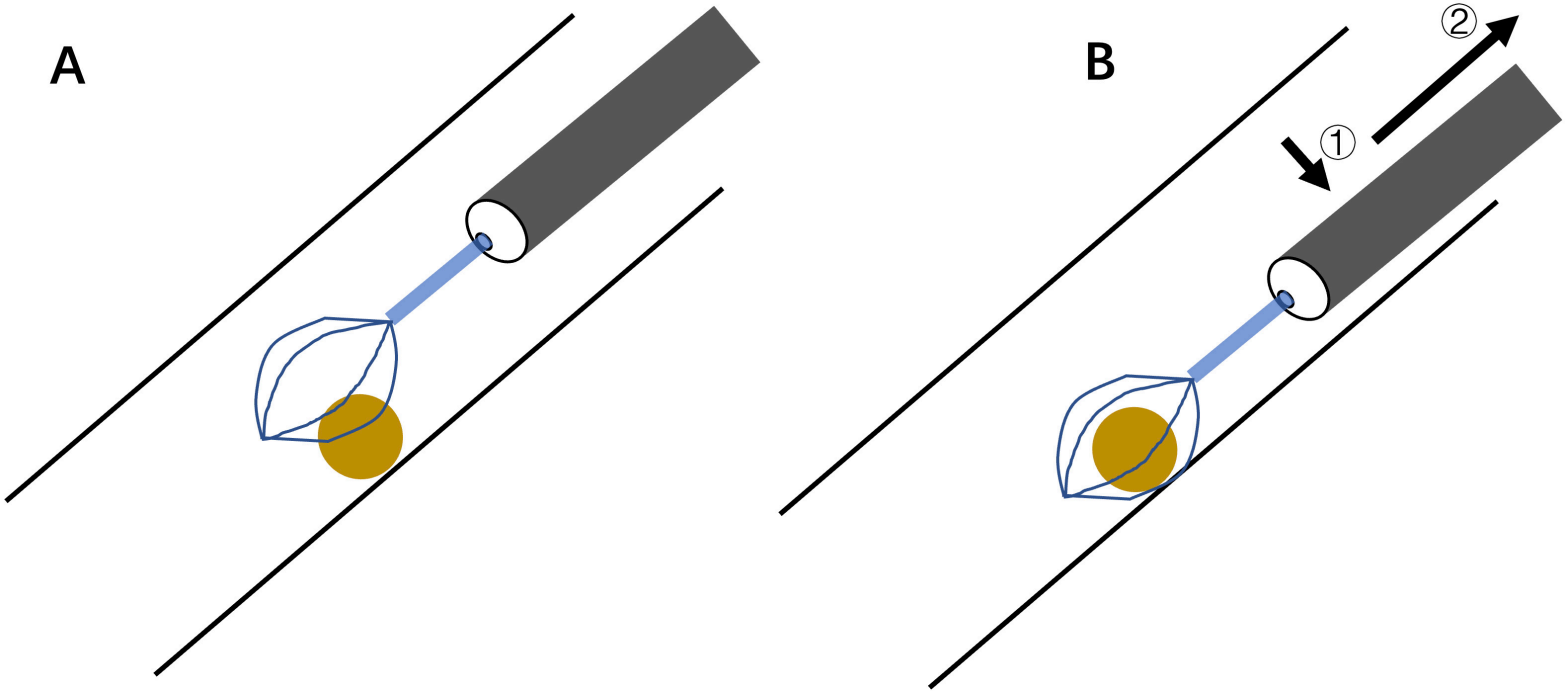
Schematic basket retrieval of small smooth/spherical AFB. **(A)** Open basket cage in line with AFB's long axis. **(B)** Advance scope to abut and cage AFB (①), and tighten cage for extraction (②). AFB, airway foreign body.

A retrieval basket is a medical tool generally comprising a flexible shaft with three or four interlocking wires that form a distal cage. Initially designed for urological stone extraction (56), its adaptation for airway interventions occurred in the 1970s, with preliminary reports documenting its efficacy in extracting small, smooth, or spherical items such as seeds or beads that resist conventional forceps retrieval (57, 58). Modern nitinol baskets have improved wire flexibility and basket-opening mechanisms, hence enhancing mobility in the distal airway (33, 59, 60). The standard technique involves advancing the closed basket through the flexible bronchoscope until the tip reaches the AFB. Then the basket is opened to form a sufficiently large spiral or diamond-shaped cage. The bronchoscope is carefully maneuvered to allow the cage to abut and encircle the AFB from its side, after which the basket is tightened to secure the object for extraction (Figure 5). The basket can be integrated with rigid bronchoscopy to enable simultaneous visualization and controlled extraction of the AFB in complex cases (61). Mild laryngeal edema has been reported in pediatric AFB retrieval with this device (33). To alleviate this complication, the AFB can be mechanically compressed along its longitudinal axis as the basket constricts, reducing its cross-sectional dimension and enabling subglottic passage. The bronchoscope along the basket-caged AFB can be removed as a single unit, similar to the conventional bronchoscopic procedure.

**Figure 6:**
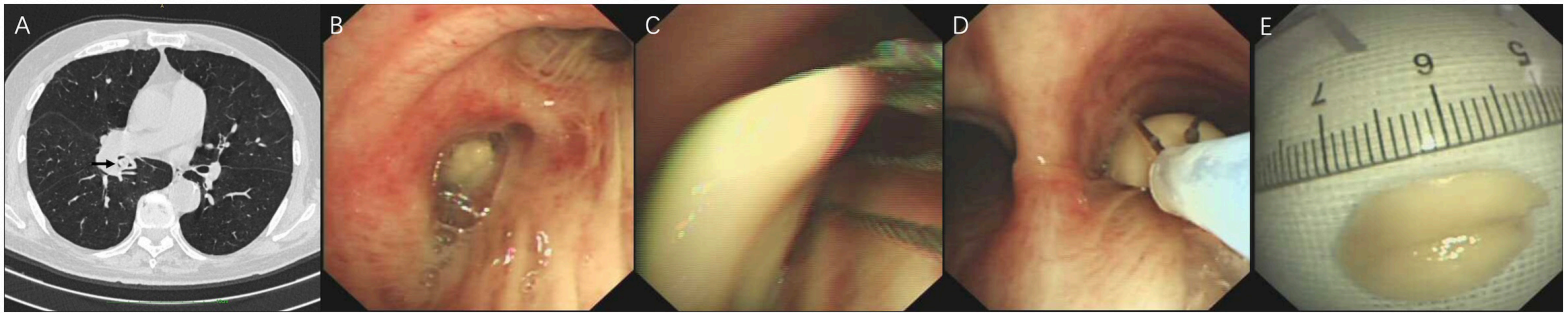
Impacted round AFB, right intermediate bronchus. **(A)** CT: low-density sphere (black arrow). **(B)** Bronchoscopy: smooth seed wedged in intermediate bronchus. **(C)** Basket opened adjacent. **(D)** Basket caged seed. **(E)** 1.5 cm peanut. AFB, airway foreign body; CT, computed tomography.

Figure 6 depicts the retrieval of a round, smooth AFB from the right intermediate bronchus. CT revealed a low-density spherical object (Figure 6A). Bronchoscopy confirmed a smooth seed wedged in the bronchus (Figure 6B). A basket was opened adjacent to the object (Figure 6C), caged the seed (Figure 6D), and extracted a 1.5 cm peanut (Figure 6E).

### Balloon

**Figure 7:**
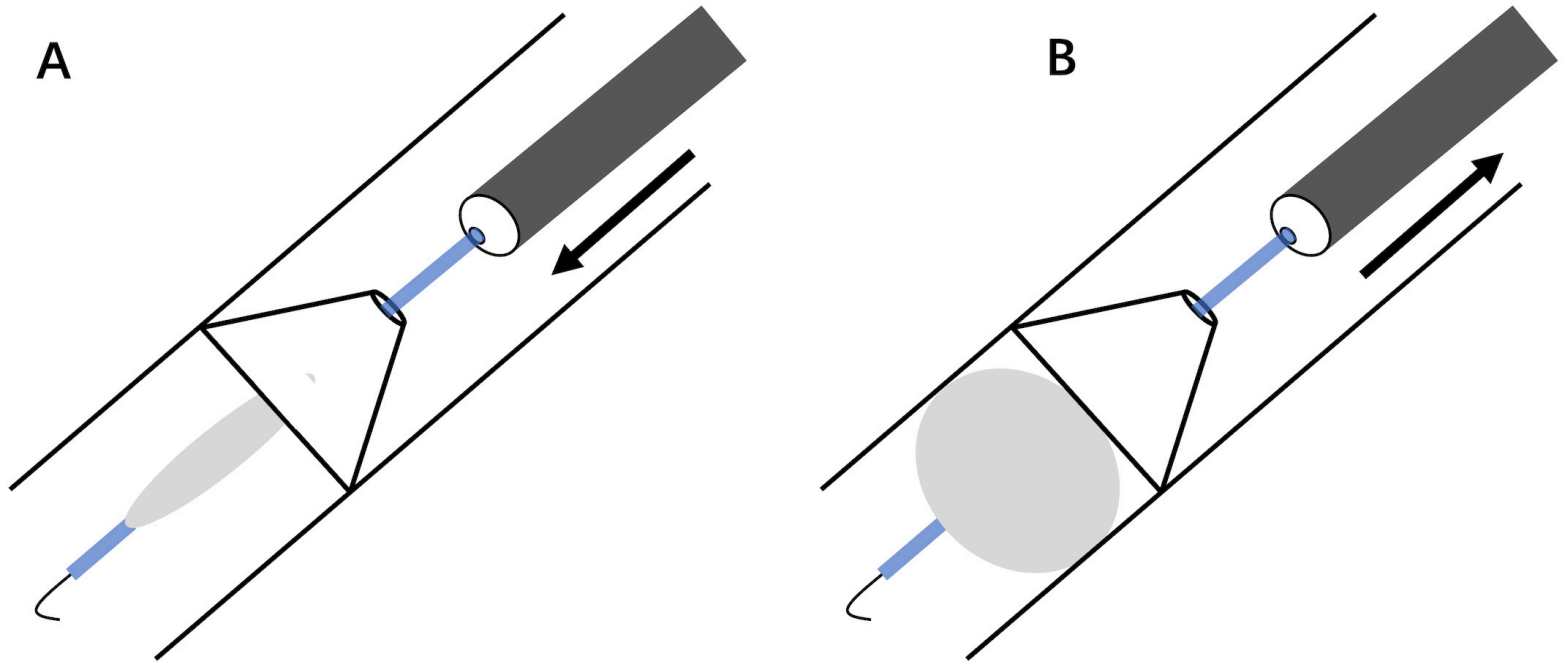
Schematic balloon retrieval of AFB with internal lumen. **(A)** Pass deflated balloon through lumen. **(B)** Inflate balloon to lock AFB for extraction. AFB, airway foreign body.

A balloon catheter is a medical device consisting of a thin tube with a saline-filled balloon at its tip, originally designed to remove blood clots from arteries and veins (62). When the AFB is embedded in the distal bronchus or defies conventional forceps, a balloon catheter may be utilized (63). For embedded AFBs with mucosal edema and granulation, the balloon catheter is initially inserted to dilate the proximal, constricted airways under flexible bronchoscopy, creating space for extraction (64). The core technique involves advancing the deflated balloon beyond the AFBs, then inflating it with saline until it gently conforms to the items. Subsequently, the inflated balloon catheter is withdrawn to bring small objects from the distal bronchus into the proximal trachea, where they can be captured and removed with grasping forceps or snares (65, 66). If the AFBs in the airway have sufficiently large lumens or openings, through which the balloon catheter can be passed and then inflated to engage the object securely for safe withdrawal (Figure 7) (67–69). The reported complications associated with balloon catheters in distal airway interventions are primarily attributed to excessive balloon pressure, including airway rupture (pneumothorax) or catheter fragmentation (70, 71). Extensive granulation tissue may impede the catheter passage, necessitating initial debridement with forceps, laser, or cryoprobe. To prevent balloon-associated complications: (1) monitor the balloon pressure within safe limits with the manometer; (2) avoid abrupt withdrawal of the inflated balloon to prevent AFB dislodgement or mucosal injury; and (3) keep the balloon catheter away from sharp-edged AFBs with a rupture risk.

### Cryoprobe

The cryoextraction technique with a cryoprobe involves the rapid formation of ice crystals triggered by the Joule-Thomson expansion of cryogenic gases (35). The freezing process facilitates the probe's adhesion to the tissue, enabling the extraction of AFBs that contain sufficient water, such as blood clots, mucus plugs, sponges, and chewing gum (34, 72–74). Based on *ex vivo* lung studies, any biologic material—including animal bones and teeth—is expected to be well-suited for cryoextraction (75). These biologic materials have rough surfaces or tiny pores that trap water or mucus, facilitating rapid ice-crystal formation and cryoextraction. Success with cryoextraction has been reported in the retrieval of aspirated teeth (76, 77).

Notably, the safety profile of cryoextraction for AFBs in adults differs substantially from that of transbronchial cryobiopsy for parenchymal lung disease (78). Pneumothorax and bleeding are frequent complications of transbronchial cryobiopsy, as the cryoprobe must be wedged into distal bronchioles—freezing in this location can lacerate airway walls and adjacent vessels (79). In contrast, the cryoextraction technique for AFB retrieval emphasizes ensuring the cryoprobe contacts only the target object while avoiding contact with normal bronchial mucosa, thereby minimizing mucosal injury and subsequent risks of pneumothorax and bleeding during the procedure. Additionally, when managing AFBs in the distal bronchi, smaller-diameter cryoprobes can be utilized. If significant resistance is encountered during extraction, it may indicate unintended contact with the bronchial mucosa; in such cases, forceful traction should be avoided, and a second attempt should be made after the ice crystals have dissolved. This tactic works perfectly for cryoextraction of chili pepper fragments, in which the probe is inserted into the pepper fragment's lumen, frozen, and then withdrawn intact (avoiding contact with normal bronchial mucosa). Severe hemorrhage is rare and typically linked to accidental cryoinjury to large bronchial vessels or underlying vascular abnormalities (80). The whole procedure needed to be performed by experts skilled in appropriate hemostatic techniques to manage severe bleeding events, such as argon plasma coagulation, laser photocoagulation, Fogarty catheter insertion, and bronchial artery embolization (81).

**Figure 8:**
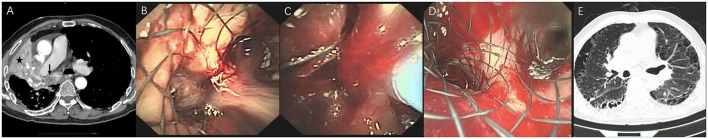
Blood clots, RMB. **(A)** CT: clot blocks RMB (black arrow); RUL collapsed (black asterisk). **(B)** Bronchoscopy: Y-stent clogged by clots at both limbs. **(C)** Cryoprobe inserted for extraction. **(D)** Y-stent fully reopened. **(E)** CT: lung re-expanded. RMB, right main bronchus; CT, computed tomography; RUL, right upper lobe.

Figure 8 illustrates the cryoextraction of blood clots in the right main bronchus (RMB). CT imaging revealed low-density clots and the collapsed right upper lobe (Figure 8A). Bronchoscopy identified the blood clots lodged in the RMB with the Y-stent (Figure 8B). A cryoprobe was inserted into the blood clots (Figure 8C), and upon freezing, the entire blood clots were extracted completely (Figure 8D). The repeated CT showed the Y-stent fully reopened and the lung re-expanded (Figure 8E).

The clinical benefits of cryoextraction often outweigh these low risks, especially for AFBs refractory to conventional tools. Compared to forceps or baskets, cryoextraction minimizes AFB fragmentation and distal embolization—complications that can lead to recurrent pneumonia, persistent airway obstruction, or the need for repeated invasive interventions (34, 74). For patients with peripherally lodged AFBs or those at high risk of desaturation (e.g., elderly individuals with comorbidities), cryoextraction via flexible bronchoscopy avoids the need for rigid bronchoscopy and general anesthesia, reducing procedural invasiveness, shortening hospitalization, and lowering the risk of anesthesia-related adverse events (44).

### Recommended algorithm

**Figure 9:**
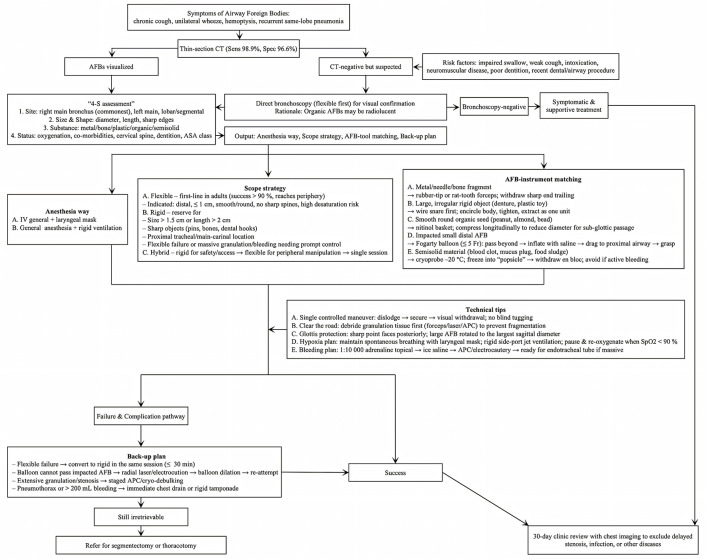
Recommended algorithm for AFB retrieval in adults. AFB, airway foreign body; CT, computed tomography; IV, intravenous; APC, argon plasma coagulation.

The recommended algorithm (Figure 9) for AFB management delineates a sequential framework commencing with symptom identification, progressing through diagnostic evaluation, risk assessment, procedural planning, and bronchoscopic intervention, accompanied by measures for addressing complications and follow-up care.

AFB in adults usually presents insidiously with cough, wheeze, or recurrent pneumonia; a history of choking is often absent, so AFB must be suspected in unexplained chronic respiratory symptoms. CT is the first-line imaging (sensitivity ≈ 99%), but negative scans do not exclude radiolucent objects and should prompt bronchoscopy. Anatomically, AFBs lodge most often in the right main bronchus. Pre-intervention planning integrates CT findings with AFB site, size, shape, substance, and patient status to select tools and determine whether to perform rigid or flexible bronchoscopy. Rigid bronchoscopy remains the gold standard for large/proximal or sharp AFBs and in small children, whereas flexible bronchoscopy via laryngeal mask anesthesia is first-line in adults because success rates exceed 90% and it accesses peripheral airways. Retrieval tools are chosen according to object properties: forceps (metal/bone), snare (large/irregular rigid items), basket (smooth/round organic seeds), balloon (small distal AFBs after dilation), and cryoprobe (semisolid water-rich material such as blood clots, mucus plugs, or food). Safe extraction requires atraumatic dislodgement, secure grasp, and controlled withdrawal; combinations of scopes and accessories are used when necessary.

### Strength and limitation

This narrative review integrates contemporary evidence and clinical experience regarding the bronchoscopic management of AFBs in adults, suggesting a pragmatic algorithm that correlates AFB characteristics (site, size & shape, and substance) and patient status with bronchoscope modality and tool selection. It also recognizes the significance of anesthesia, interventional nursing, and thoracic surgery in successful AFB retrieval, emphasizing that it requires a collaborative team effort. This paper corresponds with actual clinical conditions and improves the feasibility of implementing the proposed algorithm across diverse healthcare settings.

However, it is important to acknowledge the various limitations. The literature search was restricted to PubMed and English-language studies, potentially excluding relevant data from non-English publications or other databases. The review predominantly depends on small single-center case series, retrospective cohorts, and case reports, as there is a scarcity of large-scale prospective studies or randomized controlled trials. This heterogeneity in study design, sample sizes, and outcome measures limits the ability to conduct quantitative syntheses (e.g., meta-analyses) and may introduce selection bias. Clinical practices for AFB retrieval (e.g., anesthesia selection, tool preference, multidisciplinary team composition) vary across institutions and regions. The lack of standardized procedural protocols in included studies makes it challenging to generalize the proposed algorithm to all clinical settings. These limitations highlight the need for future prospective multicenter studies to validate the proposed algorithm and standardize the procedural workflows of adult AFB management.

## Conclusion

Adults with unexplained persistent respiratory symptoms should undergo evaluation for the presence of AFB through thin-section CT or even bronchoscopy when suspicion remains, even when imaging is negative. An individualized strategy should be guided by patient-specific factors, most critically respiratory failure status, alongside AFB characteristics and operator expertise. For stable patients without respiratory compromise, flexible bronchoscopy first and rigid bronchoscopy reserved for certain challenging cases, in combination with AFB-tailored tools, optimize retrieval success and minimize morbidity. The reliable AFB retrieval requires the availability of both flexible and rigid bronchoscopic systems, a multidisciplinary team skilled in interventional pulmonology, anesthesia, interventional nursing, and thoracic surgery, and stringent manometric monitoring through the whole procedure.
